# Comparison of three diffusion encoding schemes for cardiac imaging under free breathing conditions.

**DOI:** 10.1186/1532-429X-18-S1-W16

**Published:** 2016-01-27

**Authors:** Kévin Moulin, Alban Chazot, Jérôme Chaptinel, Pierre Croisille, Magalie Viallon

**Affiliations:** 1 grid.25697.3f0000000121724233CREATIS; CNRS (UMR 5220); INSERM (U1044), University of Lyon, Lyon, France; 2 MRI Department, Siemens Healthcare, Paris, France; 3 grid.6279.a0000000121581682Department of Radiology/CHU de Saint Etienne, University of Saint Etienne, Saint Etienne, France; 4 grid.9851.50000000121654204CIBM/Radiology Department, University of Lausanne, Lausanne, Switzerland

## Background

Diffusion cardiac imaging has been challenging due to respiratory and heart motion. Recent developments in cardiac diffusion imaging proposed more robust spin echoes encoding scheme, like Acceleration Motion Correction(AMC)(1), to tackle cardiac motion. In addition, free breathing acquisition with prospective motion correction like slice following technique (2) has been shown to reduce efficiently and significantly the scan time. In this study, the effect of breathing motion on accuracy and precision using AMC, Stjekal-Tanner (Monopolar) and Twice Refocused Spin Echo (TRSE) encoding schemes combined with slice following was evaluated on a moving phantom. In-vivo comparison was achieved in volunteers.

## Methods

Monopolar, TRSE and AMC schemes were first evaluated in-vitro: a phantom composed of 2% agar gel with different sucrose concentrations (0, 5, 10, 15%) was translated by a linear motor to reproduce the breathing motion with 4 cm of amplitude and a frequency of 0.25 hz (3). Five slices, 6 diffusion directions with b-values of 0, 15, 30, 50, 75, 100, 200, 300, 400 s/mm^2^ and 3 averages were acquired with and without motion on a 3T scanner. TE = 38, 54, 62 ms for Monopolar, TRSE and AMC respectively; TR = 5 s. Prospective motion correction was realized using a cross-pair navigator and slice following.

The three same acquisition strategies were compared on 7 volunteers. A 2-min ADC protocol was used: 5 slices, 6 diffusion directions and b-values 0, 200 s/mm^2^. Five TDs shifted every 10 ms were acquired to assess cardiac motion by PCAtMIP reconstruction (4). Monopolar, TRSE and AMC were acquired in diastole and AMC in diastole and systole with TE = 38, 54, 62 ms respectively; TR = 5s.

An objective artefact quantification was calculated related to diffusion image weighted signal (S(x,y,i)) and to S0(x,y), the non-weighted reference image.

## Results

**Figure 1:**
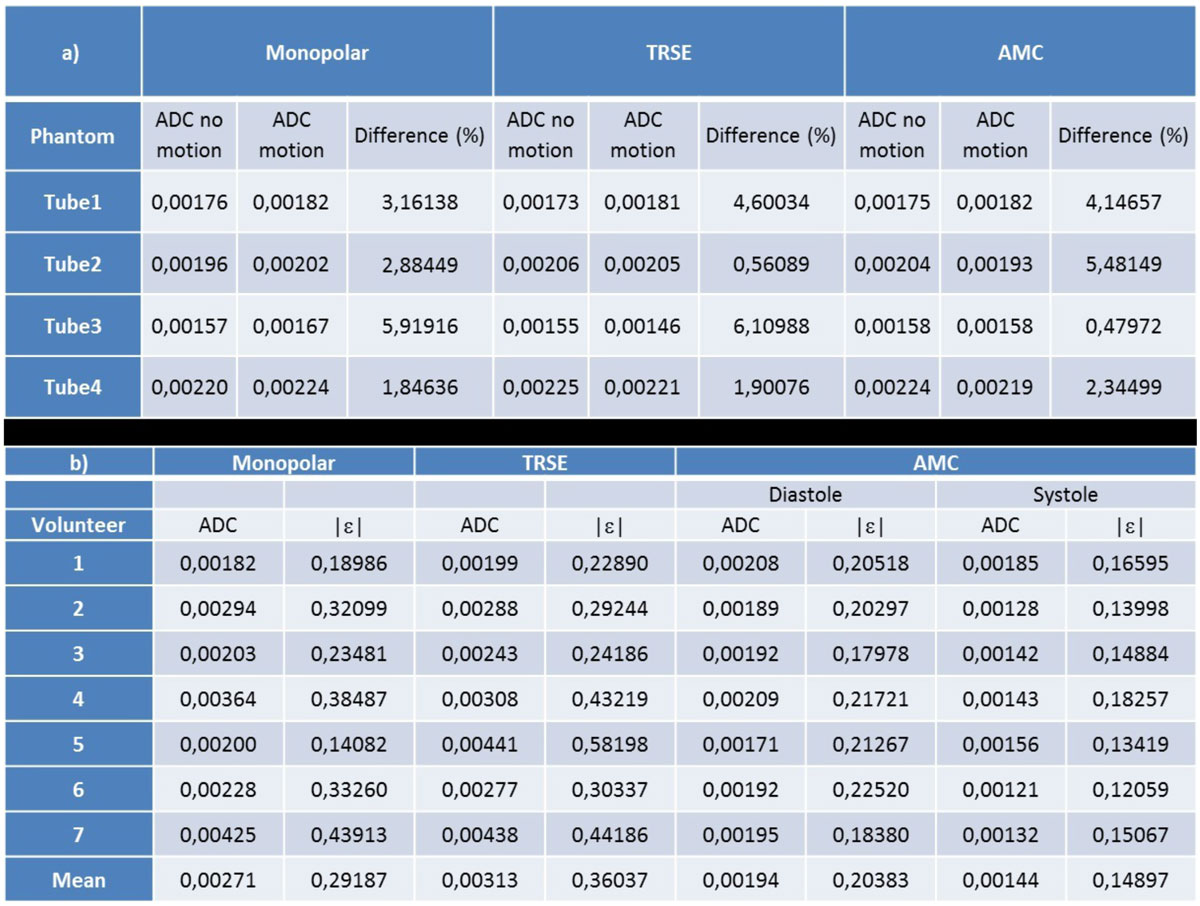
**a) ADC calculation (mm**^**2**^**/s) extracted from mono-exponential fitting on the 8 values for the 4 tubes with and without motion**. a) ADC measurement (mm^2^/s) extract from trace data and mean artifact quantification ε (no unit, mean over the 6 DWI directions) for the 7 volunteers.

**Figure 2:**
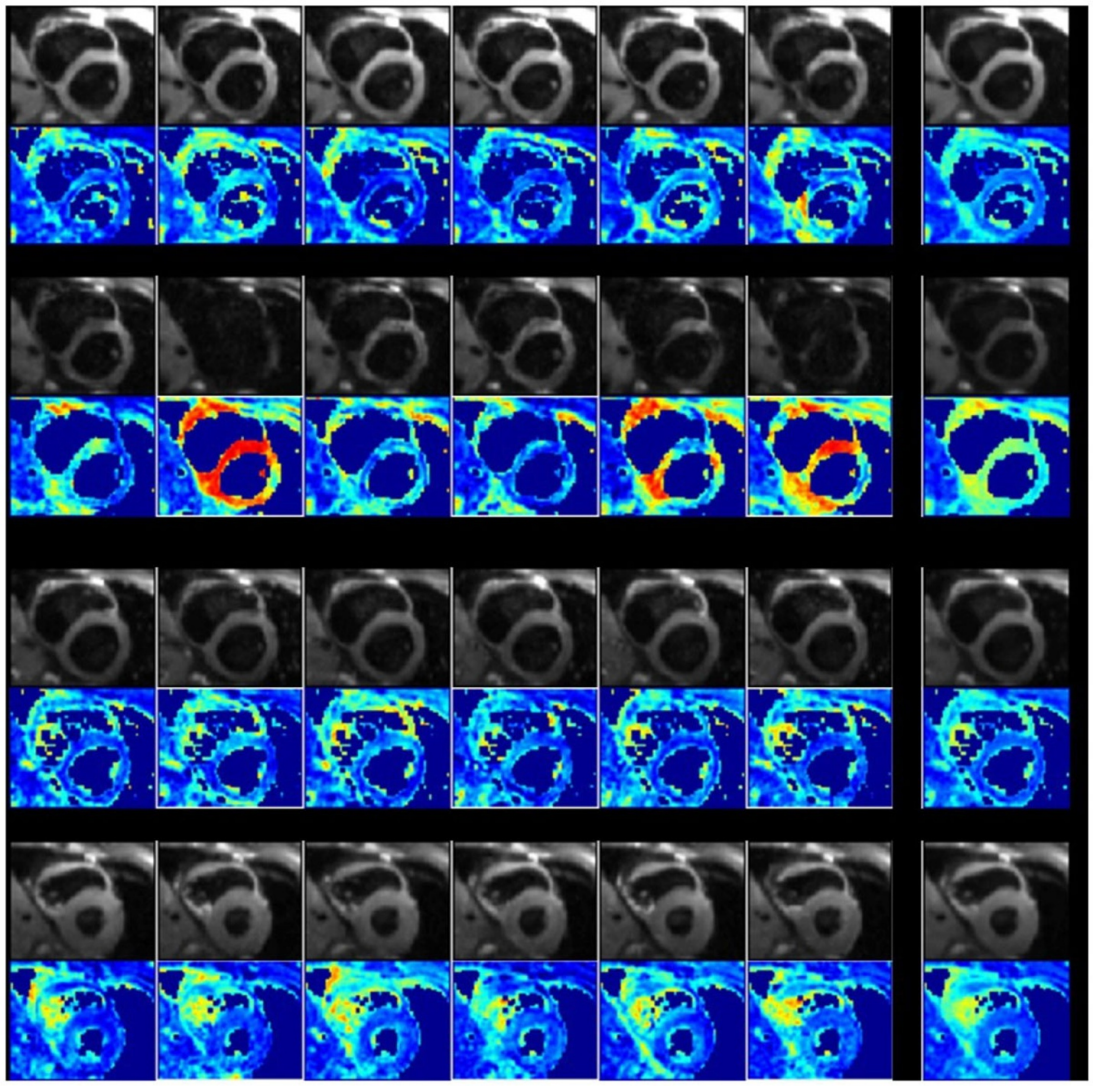
**Example of ADC raw images of mid-ventricular short axis slices**. In gray scale the Diffusion Weighted Image (DWI) for the 6 directions, b-value = 200 mm^2^/s and the Trace image. In color scale, the corresponding error map,corresponding to the artefact quantification ε. When the DWI signal drop out, artefact quantification reveal higher value.

Similar range of errors were found in the moving phantom for the three encoding scheme. The mean error was 3.5% showing that translational motion like breathing-motion affected lightly the weighting signal (Figure 1 Table a). In-vivo comparison (Figure 2) revealed a high score of artifacts for the Monopolar and TRSE encoding schemes leading to important errors in the ADC measurement: 2.71 and 3.13 * 10^-3 mm²/s respectively (Figure1 Table b). Conversely AMC appears robust to cardiac motion with low corresponding values of artefact measurement. Values of ADCs from AMC acquisition are coherent with the literature for both diastolic and systolic phase: 1.94 and 1.44 * 10^-3 mm²/s, respectively.

## Conclusions

Breathing motion compensation like slice following provides motion independent ADC estimates whatever the diffusion encoding scheme, even for ones with a longer TE (AMC, TRSE). But, cardiac and bulk motions are in opposite very critical for ADC measures, therefore they require an adequate corrected diffusion encoding scheme.
